# Matrilin-2 with a K-Chitosan Scaffold Enhances Functional Recovery and Nerve Regeneration in a Segmental Rat Sciatic Nerve Injury Model

**DOI:** 10.3390/ph18050686

**Published:** 2025-05-06

**Authors:** Neill Y. Li, Brandon Vorrius, Elliott Rebello, Jonathan Ge, Amit Mohite, Zhen Qiao, Jing Ding, Qian Chen

**Affiliations:** 1 Laboratory of Molecular Biology and Nanomedicine, Department of Orthopaedic Surgery, Alpert Medical School of Brown University, Rhode Island Hospital, Brown University Health, Providence, RI 02912, USA; brandonvorrius@gmail.com (B.V.); orthorebelmd@gmail.com (E.R.); jonathan_ge@brown.edu (J.G.); jding1@lifespan.org (J.D.); 2 Laboratory for Peripheral Neuroregenerative Biotherapeutics, Duke Nerve Center, Durham, NC 27710, USA; amit.mohite@duke.edu; 3 Division of Hand, Upper Extremity, and Microvascular Surgery, Department of Orthopaedic Surgery, Duke University Medical Center, Durham, NC 27710, USA

**Keywords:** matrilin-2, peripheral nerve repair, nerve reconstruction, reverse autograft, electrophysiology, axonal histomorphometry, collagen conduit, rat sciatic nerve, nerve regeneration, Schwann cell migration

## Abstract

**Background/Objectives:** Previous work in our lab demonstrated that a 3D scaffold containing lysine-modified chitosan (K-chitosan) and decorated with Matrilin-2 (MATN2) enhanced Schwann cell (SC) migration and axonal outgrowth in vitro and ex vivo. This study aimed to assess the regenerative effect of this scaffold compared to that of a collagen conduit and an autograft using a segmental rat sciatic nerve injury model. **Methods:** A total of 30 Lewis Rats were assigned into three groups: an untreated collagen conduit (UC) group, a collagen conduit treated with MATN2 K-chitosan (TC) group, and a reverse autograft (RA) group. Walking force measurements, compound muscle action potential (CMAP), the wet muscle weight of the tibialis anterior and the gastrocnemius, and axonal histomorphometry were assessed. **Results:** The walking force and CMAP were significantly higher in the TC group compared to those in the UC group, with no significant difference between the TC and RA groups. The muscle weights were significantly greater in the TC group compared to those in the UC group but smaller than those in the RA group. The TC group experienced significantly greater axonal regeneration compared to that with the UC, and no differences were found with the RA. The TC group further demonstrated significantly greater cell counts than those in the UC group and greater affinity of the Schwann cells towards nerve reconstruction. **Conclusion:** The MATN2 K-chitosan scaffold significantly improved nerve regeneration and was comparable to the RA, supporting the development of a novel bio-conductive scaffold conduit.

## 1. Introduction

Peripheral nerve injuries (PNIs) cause the partial to complete loss of sensory and motor function, leading to a loss of independence, chronic pain, and a significant decline in quality of life. Injury to the nerves may be caused by trauma, infection, surgical complication, compression, or autoimmune and metabolic disorders [1]. Currently, more than 20 million people in the US are estimated to suffer from some form of PNI [2]. This amounts to an annual expenditure of USD 1.1 billion on the surgical treatment of nerve injuries and USD 150 billion in overall healthcare dollars on managing these injuries [3]. Despite such costs and efforts in nerve repair and reconstruction, functional recovery, as defined by the return of motor function and sensation, occurs in less than 50% of cases [4,5]. Given these poor results, nerve surgeons are in need of innovative approaches that enhance the nerve regeneration following repair or reconstruction to enhance the reinnervation potential of the nerves and provide better, more consistent outcomes to patients sustaining such devastating injuries.

Currently, for injuries with a minimal gap, the nerves may be repaired in an end-to-end fashion as long as this is performed in a tension-free fashion. For more severe injuries in which the nerves cannot be repaired in a tension-free fashion, the use of conduits, processed allografts, and autografts is indicated, as dictated by the gap size. As conduits lack a cross-sectional architecture, their use is best supported for small gaps, commonly less than 1 cm and most commonly 5 mm [6]. The prior recommendations for conduits supported their use for gaps of up to 3 cm; however, the clinical outcomes with the use of such long distances demonstrated poor results, and thus, they have been limited to shorter distances [7]. For longer gaps, processed allografts or autografts are most frequently used. Processed allografts provide the native cross-sectional and longitudinal architecture of the nerves, but the processing of these nerves to handle immunogenic concerns also removes cells, signaling proteins, and extracellular matrix (ECM) proteins which could be key to regeneration [8]. As a result, the cells and proteins needed to initiate and sustain a regenerative microenvironment are significantly reduced. Autografts deliver both the appropriate architecture and the cells, growth factors, and ECM proteins needed for regeneration. However, their use requires a donor site—adding to the risk of hematoma, wound healing and infection complications, the loss of nerve function in the donor, and the risk of neuroma generation—and a period of Wallerian degeneration to prepare the autograft to become an appropriate regenerative construct [9]. Given the pros and cons of each of these modalities, our lab sought to create a graft predicated upon a readily available biomimetic multi-channel cross-sectional graft that harbored chemotactic and regenerative properties while bypassing donor site morbidity in order to guide the cellular migration and axonal regeneration best. Our goals in this were to advance the current indications in terms of the appropriate gap distance in conduit use for nerve reconstruction without having to explore allograft and autograft options given the costs, biological utility, operative times, and donor site morbidity associated with these approaches.

In developing this graft, Matrilin-2 (MATN2), a protein from the matrilin family of ECM proteins, which has been shown to enhance Schwann cell (SC) migration and axonal outgrowth, was integrated into chitosan, a bioactive and biodegradable polysaccharide, with lysine augmentation to form a bioactive multi-channel scaffold [10,11,12,13,14,15]. In vitro testing of this scaffold in our lab found a significant increase in SC adhesion and migration compared to these properties when using a conduit [16]. A subsequent ex vivo study using dorsal root ganglions demonstrated greater neuronal adhesion and axonal outgrowth when using this scaffold [16]. Expanding upon our in vitro and ex vivo results, the purpose of this study was to evaluate the MATN2- and lysine-enhanced chitosan (K-chitosan) scaffold in a rat sciatic nerve defect model and compare the functional and regenerative outcomes to those when using a clinically used conduit and an autograft. We hypothesized that the implementation of the biomimetic scaffold, which enhances Schwann cell adhesion and migration and is supportive of axonal outgrowth, would offer better functional, electrophysiological, and histological outcomes than those with a conduit and would be comparable to those with an autograft.

## 2. Results

### 2.1. Functional Outcome Measurements

The 3D scaffold of matrilin-2 with K-chitosan in a collagen conduit was prepared as described previously [16]. The conduit and the conduit with the scaffold were applied to rat sciatic nerve reconstruction. A gait analysis, as well as measurements of compound muscle actional potential, were used to measure the functional recovery following reconstruction as follows:

### 2.2. Analysis of Walking Track Force

**Figure 1:**
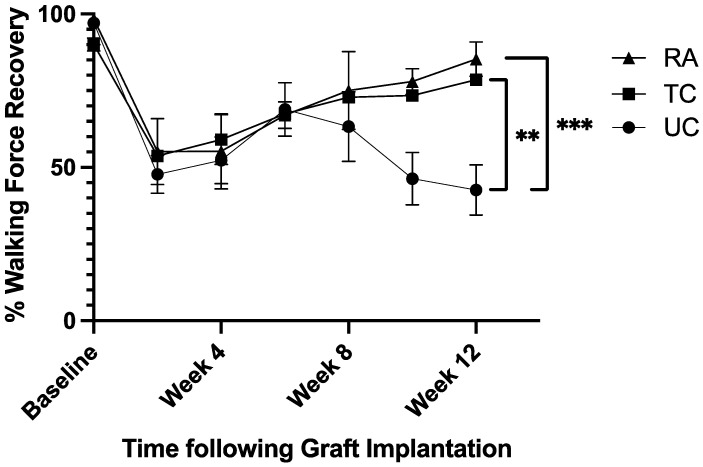
Walking force on experimental left hindlimb in proportion to that on normal right hindlimb. Measurements taken preoperatively and every 2 weeks postoperatively for 12 weeks. RA: reverse autograft; TC: treated conduit; UC: untreated conduit. ** *p* ≤ 0.01, *** *p* ≤ 0.001. Error bars represent the standard deviation (SD) in the data, with *n* = 8 for each group.

The walking track gait analysis evaluated the proportion of force placed on the treated left hindlimb (LHL) compared to that on the control right hindlimb (RHL). The baseline values averaged 95.1% ± 4.5% across all three groups. Following sciatic nerve reconstruction, the walking force fell to an average of 52.2% ± 3.9% following surgery. The untreated conduit (UC) group fell to 42.6% ± 8.2% by week 12 (Figure 1), while the treated conduit (TC) and reverse autograft (RA) groups steadily rose to 78.5% ± 1.4% and 85.2% ± 5.6%, respectively (Figure 1). The analysis of the week 12 force measurements found significant differences between the UC and TC groups (*p* = 0.0035) and the UC and RA groups (*p* = 0.001) (Figure 1).

### 2.3. The Compound Muscle Action Potential (CMAP)

**Figure 2:**
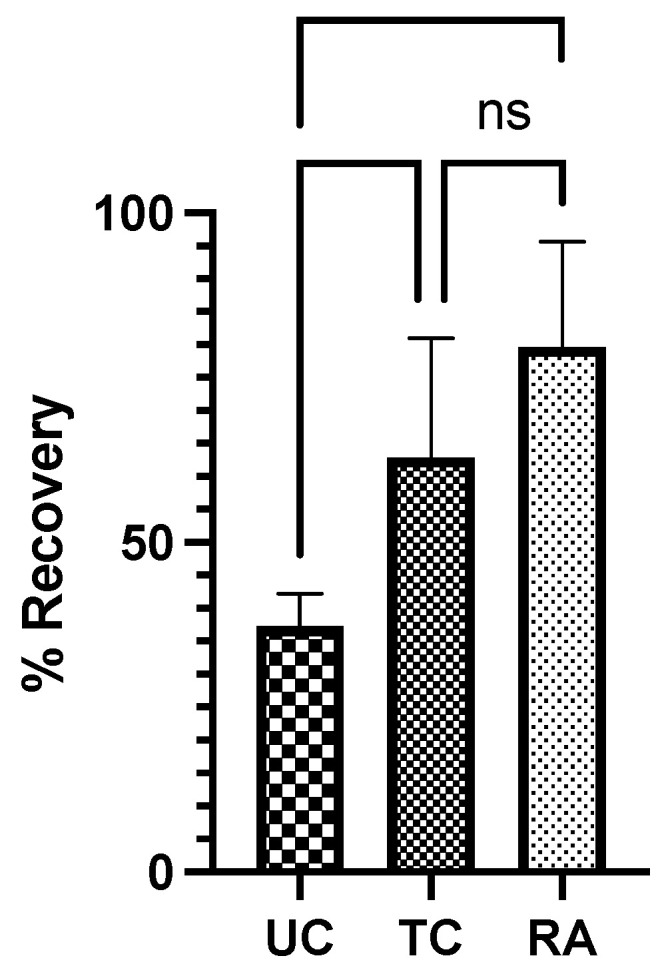
The compound muscle action potential was recorded for the experimental left hindlimb and the control right hindlimb for each rat and normalized as the proportion of recovery for each rat. UC: untreated conduit; TC: treated conduit; RA: reverse autograft. * *p* ≤ 0.05, ** *p* ≤ 0.01, ns: not significant. Error bars represent the standard deviation (SD) in the data, with *n* = 8 for each group.

The CMAP was evaluated in the gastrocnemius of each rat to calculate the proportion of sciatic recovery in the experimental LHL compared to the control RHL. The UC group demonstrated a mean recovery of 37.3% ± 4.95% compared to the normal RHL. Comparatively, the TC group had 62.9% ± 18.1% recovery, and the RA group had 79.7% ± 15.9% recovery (Figure 2). Significant differences in the proportional recovery were found between the UC and TC (*p* = 0.044) and UC and RA groups (*p* = 0.0013). No difference was found between the TC and RA groups (Figure 2).

### 2.4. The Weights of the Tibialis Anterior and Gastrocnemius Muscles

**Figure 3:**
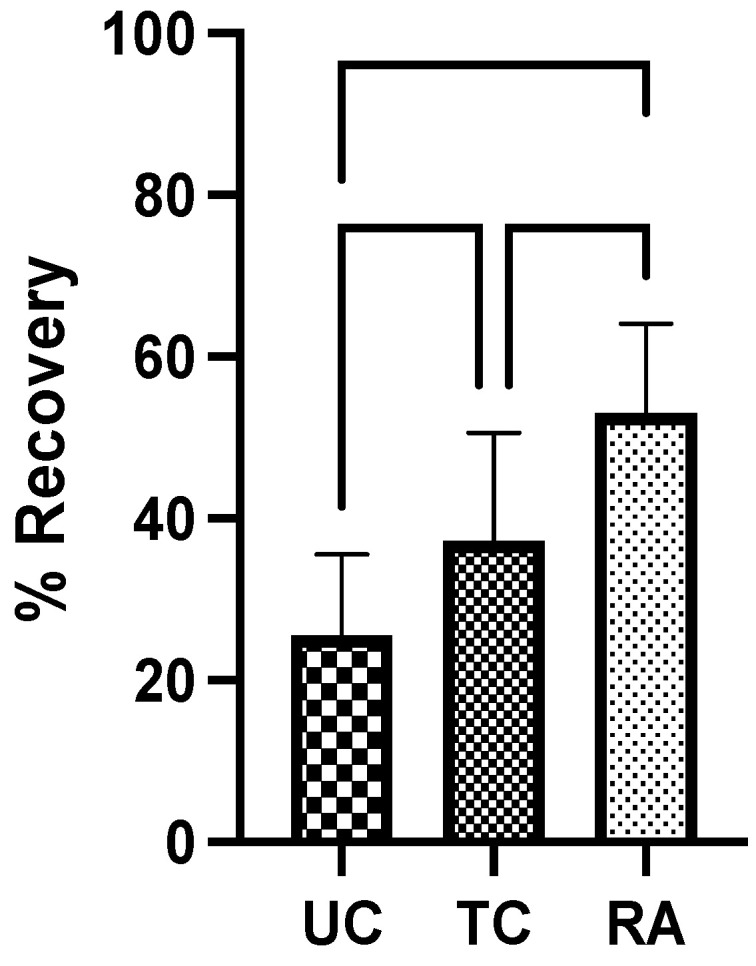
Muscle weights of tibialis anterior and gastrocnemius from experimental left hindlimb normalized to those in right hindlimb to generate proportion of recovered muscle mass after 12 weeks. UC: untreated conduit; TC: treated conduit; RA: reverse autograft. * *p* ≤ 0.05, ** *p* ≤ 0.01, **** *p* ≤ 0.0001. Error bars represent the standard deviation (SD) in the data, with *n* = 8 for each group.

The TA and GSC muscle weights in the experimental LHL were normalized to those in the RHL determine the percentage of muscle in the LHL that remained and the level of denervation atrophy. The UC group had the lowest weight of preserved muscle at 25.6% ± 9.93%, followed by that in the TC group at 37.2% ± 13.4%. The RA group had the greatest proportion of remaining muscle at 52.9% ± 11.1%. Significant differences in muscle weight were identified between the UC and TC groups (*p* = 0.0202) and the TC and RA groups (*p* = 0.0030) (Figure 3). The most notable difference, however, was noted between the UC and RA groups (*p* < 0.0001) (Figure 3).

### 2.5. Axon Quantification

**Figure 4:**
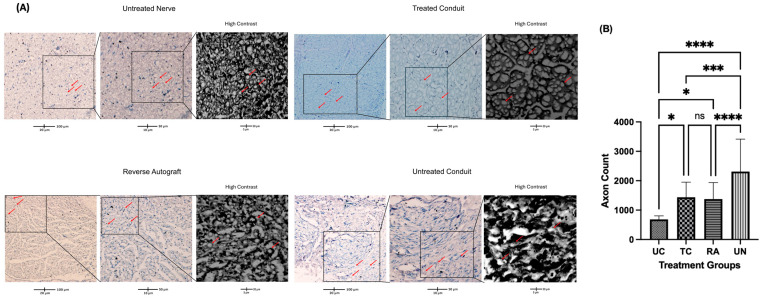
The number of regenerated axons in the distal nerve stump following sciatic nerve reconstruction. (**A**) Cross-sections of toluidine-blue-stained nerves immediately distal to the conduit or the autograft segment in the UC, TC, RA, and UN groups. (**B**) Quantification of the mean axon number for each nerve in the RC, TA, RA, and UN groups. Red arrows indicate examples of counted axons. Scale bar of 100 µM. * *p* ≤ 0.05, *** *p* ≤ 0.001, **** *p* ≤ 0.0001, ns: not significant. UC: untreated conduit; TC: treated conduit; RA: reverse autograft; UN: uninjured nerve. Error bars represent the standard deviation (SD) in the data, with *n* = 8 for each group.

The numbers of regenerated axons were visualized through toluidine blue staining and counted at the distal nerve stump immediately distal to the conduit and the autograft reconstruction suture sites. This ensured consistency in capturing the number of axons that were able to successfully traverse the segmental defect and comparing them across native nerves. Each identified axon was counted for the entirety of the cross-section of each nerve. Grossly, the density of the axons in the UC group was reduced compared to that in the TC, RA, and uninjured (UN) groups (Figure 4A). The lack of axonal density in the UC group was met with a greater degree of disorganized connective tissue. Comparatively, the TC group demonstrated more connective tissue organization and a higher axonal density. A quantitative analysis of the axon number revealed a mean of 686.0 ± 117.4 axons for the UC group, 1438 ± 514.5 axons for the TC group, 1374 ± 565.2 axons for the RA group, and 2270 ± 1103 axons for the UN group (Figure 4B). Significant differences in axon number were counted between the UC and TC (*p* = 0.0331), UC and RA (*p* = 0.0433), UC and UN (*p* < 0.0001), TC and UN (0.0019), and RA and UN (*p* = 0.0002) groups. No significant differences were noted between the TC and RA groups (*p* = 0.9939).

### 2.6. Schwann Cell Immunofluorescence Differential Distribution

**Figure 5:**
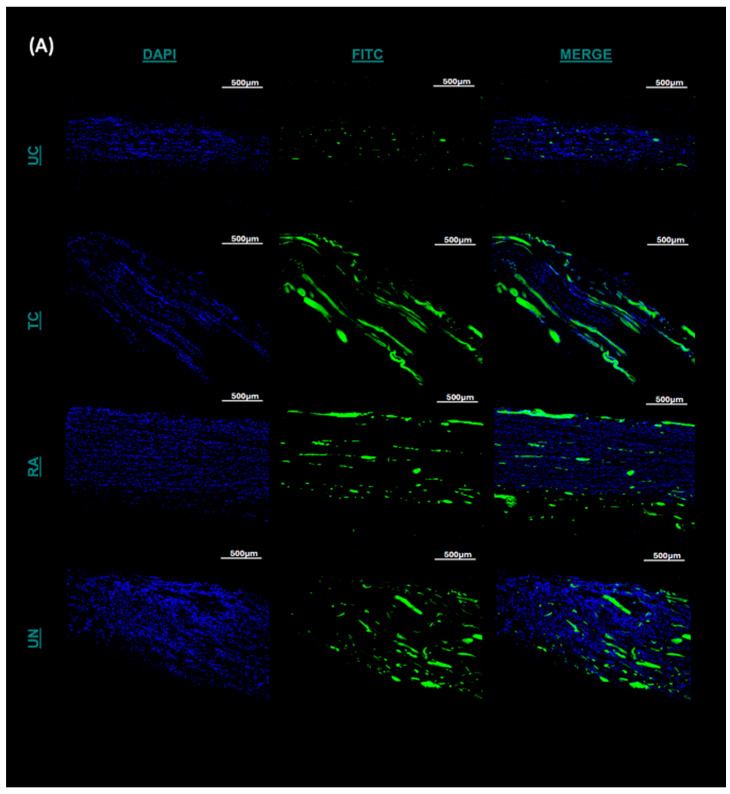

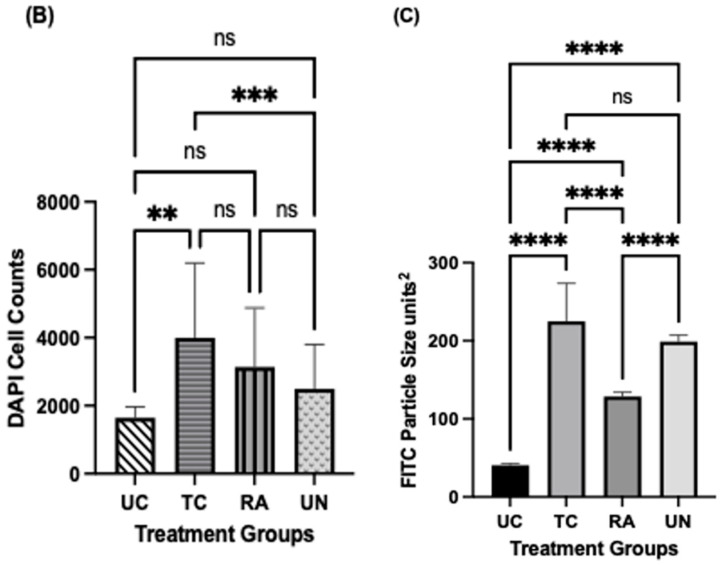
Immunofluorescence staining of the longitudinal section of the distal extent of the reconstructed nerve segment. (**A**) Green fluorescence represents Schwann cells and blue fluorescence represents DAPI. Scale bar at 500 µM. UC: untreated conduit, TC: treated conduit, RA: reverse autograft, UN: uninjured nerve. (**B**) Quantification of mean DAPI fluorescent for cell number of each nerve at 4× magnification. (**C**) Measurement of mean particle size of Schwann cell fluorescent signal for each nerve at 10× magnification. Scale bar of 100 µM. ** *p* ≤ 0.01, *** *p* ≤ 0.001, **** *p* ≤ 0.0001, ns: not significant. Error bars represent the standard deviation (SD) of the data, with *n* = for each group.

Immunofluorescent staining was utilized to evaluate the presence and distribution of Schwann cells (SCs) within the untreated and treated conduits and the autografts in the reconstructed sciatic nerves at their most distal points. Between the UC and TC imaging, there was greater cross-sectional conglomeration of the SCs and DAPI-positive cells within the TCs than the UCs (Figure 5A). The SC distribution with the UC was contained to a central localized area within the empty conduit rather than occurring across the entirety of the cross-section (Figure 5A). More structured longitudinal and cross-sectional populations of SC fluorescence were visualized with the TC compared to those with the UC, and they were more confluent compared to those with the RA (Figure 5A). When quantifying the cell number using DAPI, significant differences were noted between the UC, TC, and RA groups. The TC group demonstrated significantly greater cell counts than those in the UN (*p* = 0.00082) and UC (*p* = 0.0072) groups (Figure 5B). Furthermore, the TC group demonstrated higher affinity towards clustering and propagation of the SCs than these properties in any other treatment group, as noted from the larger fluorescent particle sizes measured in Figure 5C. Significant differences were noted for the TC group between the TC and UC (*p* = 0.0064) groups and the TC and UN groups (*p* = 0.0003). No difference was noted between the TC and RA groups. Together, such findings demonstrate the chemotactic potential of the matrilin-2/k-chitosan scaffold given the high SC counts. The larger particle sizes noted within the TC group further supported that the migration and adhesion in Schwann cell chemotaxis facilitated a more normalized nerve microenvironment, as seen in an uninjured nerve.

## 3. Discussion

In this study, we evaluated the in vivo impact of a MATN2 and K-chitosan scaffold on the regeneration of segmental defects in rat sciatic nerves compared to that with no scaffold and the use of an autograft. The rats from each group were tested functionally using gait trials, from which we found that the restored paw pressure in the TC group was greater than that in the UC group and equivalent to that in the RA group at 12 weeks. Prior to their sacrifice, the conduction of the nerve to the gastrocnemius was assessed through the CMAP, revealing greater conduction and activation of the gastrocnemius with the TC compared to that with the UC and comparable conduction and activation to that with the RA. The muscle weights demonstrated the least atrophy in the RA group and significantly less atrophy in the TC group compared to that in the UC group. Finally, the number of regenerated axons was quantified at the distal stump, with a greater number of regenerated axons noted in the TC group compared to that in the UC group, with no significant differences from the results with the RA. An immunofluorescence evaluation of the quantity and distribution of the SCs noted a greater spatial distribution and quantity in the TC group compared to those in the UC group, with equivocal findings to those with the RA. The inset of the Matrilin-2/K-chitosan scaffold into a commercial collagen conduit significantly increased the functional, electrophysiologic, and histological measures of nerve regeneration compared to those when using the hollow conduit. In comparison to those with the RA, functional measures such as paw pressure in the walking track analysis and muscle innervation in the evaluations of the CMAP, as well as the axonal counts and Schwann cell profiles, were comparable. These findings support the regenerative benefit of the readily available matrilin-2- and lysine-enhanced chitosan scaffold in advancing Schwann cell migration and axonal regeneration comparably to a reverse autograft following segmental nerve injury and providing biological enhancement to support the potential reconstruction of mid-length nerve defects in clinical practice, which is not currently available in the US.

Following a nerve injury that requires segmental reconstruction, successful regeneration occurs as the axons from the proximal nerve stump are able to traverse the defect and reach their respective muscle and sensory targets. A crucial mechanism for this to occur resides in the Schwann cells and their migration ability. Once these cells have myelinated, they need to dedifferentiate into reparative cell phenotypes and begin to migrate across the injury site to facilitate the formation of the bands of Büngner that ultimately provide the guide for axonal outgrowth [17]. For this to occur, the microarchitecture of the extracellular matrix and its composition are major factors implicated in supporting the migration of the Schwann cells. The inner composition of nerves consists of a complex network of factors, including collagen, laminin, fibronectin, and matrilin-2, that situate and physically suspend regenerative cellular components [18,19]. As pertains to nerve regeneration, this microarchitecture offers contact forces, both attractive and repulsive, which affect the migration of the Schwann cells and the chemotaxis of immune cells along the length of nerves [20]. A major deficit in basic conduit repair is the lack of a cross-sectional microstructure to support the regenerating axons, as well as the reliance on a fibrin clot to support regeneration, which is at risk of disruption.

Studies have demonstrated that the cross-section of autografts and their microarchitectures consistently outperform empty collagen conduits, thus compelling continued investigation into improving off-the-shelf devices to limit the morbidity associated with autografts [8,21].Thus, formulating a biomimetic microarchitecture has recently become a major point of study in peripheral nerve regeneration. Many intra-conduit structures have been tested in terms of their impact on neurite outgrowth, including carbon nanotubes, embedded axons, growth factors, and even Schwann cells [22,23,24]. These challenges highlight the need for innovative biomaterial scaffolds capable of not only providing a structural construct but enhancing the regenerative properties further to permit healing across longer gaps. Our novel K-chitosan scaffold with added MATN2, formed by crosslinking chitosan and lysine and mixing in added MATN2 using sonication, offers improved bioactivity and mechanical strength over those of conventional chitosan. Lysine provides additional integrin-binding motifs that enhance Schwann cell adhesion and migration. When combined with matrilin-2, a matrix protein known to stabilize the extracellular architecture and promote axonal regrowth, this composite scaffold addresses the gaps in peripheral nerve repair. No studies have combined these elements into a unified conduit system, which offered us a significant opportunity. In this study, our novel approach, beyond the interest to seed scaffolds with cells or growth factors, was the combined use of MATN2, an extracellular matrix protein, and chitosan, a natural biopolymer, with each having strong regenerative properties, in the peripheral nerves to create a porous biomimetic extracellular matrix based scaffold.

The derivation of this scaffold was centered upon the biomimetic influence of matrilin-2 (MATN2), previously shown to allow for greater Schwann cell migration and adhesion, as well as axonal outgrowth, in dorsal root ganglion assays through potential integrin mediated pathways [10,11,16,25,26]. Matrilin-2 is a highly expressed protein in extracellular matrices throughout the body, including the skin, cartilage, bone, uterus, and many other sites [27]. It has been found to be crucial and necessary for peripheral nerve regeneration to occur, with Malin et al. finding delayed regrowth and functional recovery in MATN2-deficient mice [11]. In malignant pilocytic astrocytoma, MATN2 was upregulated, further suggesting its specific contribution to nerve growth [28]. One of the main functions of MATN2 is to modulate the collagen in the ECM, interacting with itself and other matrix proteins to create orderly networks of elongated fibrils [13]. These fibrillar structures offer structural and biological incentives for axonal outgrowth, as seen in the ideal porosity for the attractive forces, as well as the ideal cell adhesion molecules, for the migration of catecholamine-producing PC12 cells. MATN2 possesses uniquely beneficial properties for its inclusion in scaffolds for nerve regeneration [29,30]. Prior in vitro studies have shown MATN2 to perform better than other ECM components, namely fibronectin and laminin, in stimulating SC migration [11]. Mechanistic studies into understanding the underlying processes behind these improvements are limited, and future work is required to delineate them further. Nonetheless, given the specific favorable characteristics of MATN2 in enhancing Schwann cell and axonal behavior, its presence within the scaffold offers a biomimetic environment that enhances a more robust regenerative response.

Chitosan is a polysaccharide-based biopolymer that has been studied for its potential to create a porous environment like that in the native ECM [31]. It has beneficial effects on axonal outgrowth, including cell adhesion, biodegradation, and biocompatibility. Chitosan is a polycationic polysaccharide whose charge gives it bioactivity in associating with the anionic electrolytes and anionic regions in cell membranes, as well as an antimicrobial effect in disrupting bacterial cell walls [32,33]. As a biopolymer, chitosan can be naturally degraded by native enzymes, creating byproducts known as chitooligosaccharides (COSs). These COSs stimulate the production of macrophage-attracting factors by the SCs such as chemokine ligand 2, which, in turn, attracts M2-polarized macrophages to the site of injury, which induces repair changes in the tissue microenvironment through IL-10 and IL-13 secretion [34,35]. As a result of these properties, chitosan has been used in a variety of methods for nerve regeneration [36,37,38] Engineered chitosan matrices with enhanced implementations of nanofibers and microchannels have been shown to provide a beneficial porosity and surface-area-to-volume ratio for the appropriate traversal of inflammatrrrrrrory factors, SCs, and axon sheaths [36,37,38]. Chitosan conduits have been created and seeded with varying use of the muscle, hydrogels, and deacetylation profiles and varying outcomes in rat sciatic nerve models [39,40,41]. One clinical study demonstrated improved sensory outcomes in digital nerve reconstruction utilizing chitosan-based conduits [12]. Commercially, chitosan is also being used as the sole material for the nerve wraps and conduits distributed by Checkpoint Surgical and Kerimedical in the United States, as well as Europe. No long-term adverse effects of chitosan when utilized on nerves have been reported, nor have they for any applications where chitosan is applied in either supplementation form or device use [42]. Growing presence and use of this device are seen for the United States, alongside growing familiarity with chitosan as a biomaterial in Europe [43]. Unique to this study was the modification of the amine group with the addition of lysine in order to enhance the cationic character of the biopolymer to ultimately enhance the cellular and protein affinity. The use of the lysine-enhanced chitosan allowed the cross-sectional and longitudinal presence of MATN2 to provide a chemotactic enhancement in SC migration and axonal regeneration. This technique reproduced the positive results of other studies of chitosan-based scaffolds, with comparable results to functional benefits of autografts. There remains a need for future studies that elucidate the mechanistic and immune-related differences with the combined use of MATN2 and K-chitosan [40,44].

Following nerve reconstruction, nerve and muscle functions are goal-oriented parameters for analyzing the rate and quality of regeneration. In this study, we assessed (1) muscle function using an automated gait analysis trial, (2) nerve function through the CMAP, and (3) muscle restoration using muscle mass measurements [45,46,47,48]. In the walking trial, we found that the rats in the TC group exhibited greater use of their reconstructed hindlimbs compared to that with the UC, suggesting increased axonal regeneration and passage of signals through the MATN2/K-chitosan scaffold. No differences were found between the RA and TC at 12 weeks, suggesting that the TC promoted enough axonal regeneration to support a greater functional recovery than that with the UC. Secondly, the assessment of the nerves through the CMAP quantifies the functional recovery of the axons through the reconstruction site to support muscle function. We found that the RA provided an increased signal amplitude and frequency compared to those with both the TC and UC, with the TC performing better than the UC. This suggests that the presence of the matrilin-2- and lysine-enhanced chitosan scaffold allows for greater functional axonal regeneration in the muscle targets compared to that with empty conduits. Finally, the weight of the gastrocnemius and tibialis anterior complexes at the end of the study period, marking the degree of denervation atrophy, revealed that the most muscle was preserved with the autograft reconstruction [49]. However, the rats that underwent matrilin-2- and lysine-enhanced chitosan scaffold reconstruction demonstrated greater muscle preservation than that in those with conduit reconstruction, further supporting Schwann cell migration and axonal regeneration through the reinnervation of the muscles.

Cross-sections of the distal nerve stumps from the reconstructed zones after the study period revealed variance in the microarchitecture, axon counts, and myelination. The conduit reconstruction demonstrated large gaps between the axons and a poorly developed microarchitecture overall owing to the decreased cross-sectional support in the conduit environment. In accordance, the quantity and distribution of the SCs under immunofluorescence were reduced and more narrowly centralized in the conduit group. In contrast, the TC group demonstrated a greater number of axons compared to that in the UC group. In addition, the immunofluorescence evaluation demonstrated greater cell numbers and Schwann cell fluorescence with the TC compared to those with the UC. When comparing the TC against the autograft and normal nerves, there was a significantly greater number of axons in the normal nerves but no statistical difference when comparing the TC group to the autograft group. On fluorescence evaluation, the Schwann cell confluence, migration, and propagation were seen to be the best in distribution between the use of the TC and UN, with quantification of the fluorescence, numbers, and size indicating no significant difference between these two groups. These results further exemplify the crucial role and presence of the SCs in cultivating a microenvironment that is most conducive to regeneration and the restoration of normal physiology [36]. Thus, the presence of MATN2 and chitosan in the biomimetic scaffold to enhance SC chemotaxis is a crucial attribute in its development, as is the inclusion of the chitosan backbone, with its beneficial properties for macrophage chemotaxis having previously been demonstrated [11,16]. Together, the chemotactic and biomimetic potential of this scaffold permits a more robust cellular response to best enhance peripheral nerve regeneration and functional recovery.

One limitation of this study is that it was limited to a 12-week-interval assessment. Given its beneficial effect in the 12-week period, future studies are needed to advance to longer-term metrics for one year and two years following implantation to delineate longer-term impact of the scaffold’s presence. Long-term studies could evaluate the differences in the immune response further by understanding the differences in the presence and behavior of macrophages, fibroblasts, T-cells, and Schwann cells, as well as chemokine and cytokine protein profiles, based on histological and protein quantification assessments using multiplex ELISAs. An additional limitation is the need to further understand the differences in the number and morphology of neuromuscular junctions, as well as the health of the neurofilament interactions. This could be assessed further through NMJ staining and neurofilament staining between groups.

## 4. Materials and Methods

### 4.1. The Preparation of Matrillin-2

**Figure 6:**
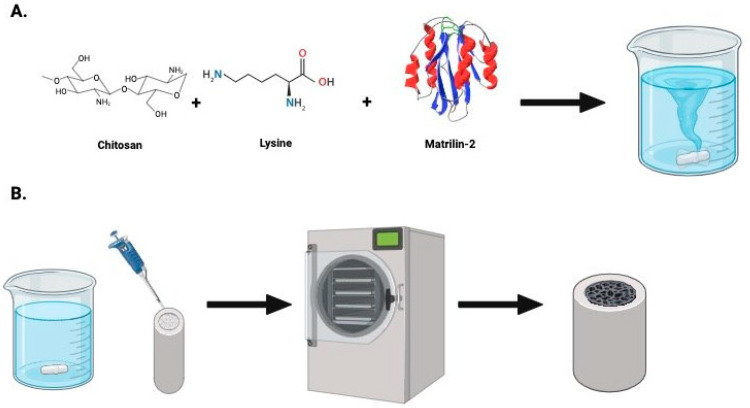
A schematic of the development of the lysine- and matrilin-2-enhanced chitosan. (**A**) The initial preparation of chitosan and lysine. After crosslinking the lysine and chitosan, matrilin-2 was added to create a homogenous gel. (**B**) The gel was then placed into a collagen conduit, which was freeze-dried and then lyophilized to create a porous matrilin-2-/lysine-enhanced chitosan scaffold housed in a collagen conduit.

The preparation of our matrilin-2-/lysine-enhanced chitosan scaffold was previously described by us [16]. In brief, low-molecular-weight chitosan (Sigma-Aldrich, cat. No. 448869, St. Louis and Burlington, MA, USA) was dissolved at 2% (*w*/*v*) in 1% (*v*/*v*) acetic acid solution and stirred at room temperature to form a homogenous gel. A lower molar ratio of lysine was then added to bind the free amine groups on the chitosan chain and the mixture stirred again to form a homogenous gel once more. *N*,*N*’-Dicyclohexylcarbodiimide (DCC) was then added as a coupling reagent to trigger a reaction, followed by dialysis to remove the DCU byproduct. Glutaraldehyde was added to facilitate crosslinking between the lysine and chitosan. Upon completion, matrilin-2 was added using a displacement pipette and mixed through continuous pipetting until homogeny was achieved. The gel was then pipetted into a type 1 collagen conduit (Integra Lifesciences, Princeton, NJ, USA) and lyophilized until a dry, porous structure formed (Figure 6).

### 4.2. Animals

All of the animal experiments and procedures were performed in strict accordance with the National Institute of Health Guidelines for Laboratory Animal Care and Safety, with the protocols approved by the Lifespan Animal Welfare Committee (Institutional Animal Care and Use Committee No. 500220). Male adult Lewis Rats (220–250 g) were purchased (Charles River Laboratories, Wilmington, MA, USA) and given ad libitum access to water and food over a 12 h light/dark cycle.

### 4.3. The Sciatic Nerve Defect and Reconstruction Models

**Figure 7:**
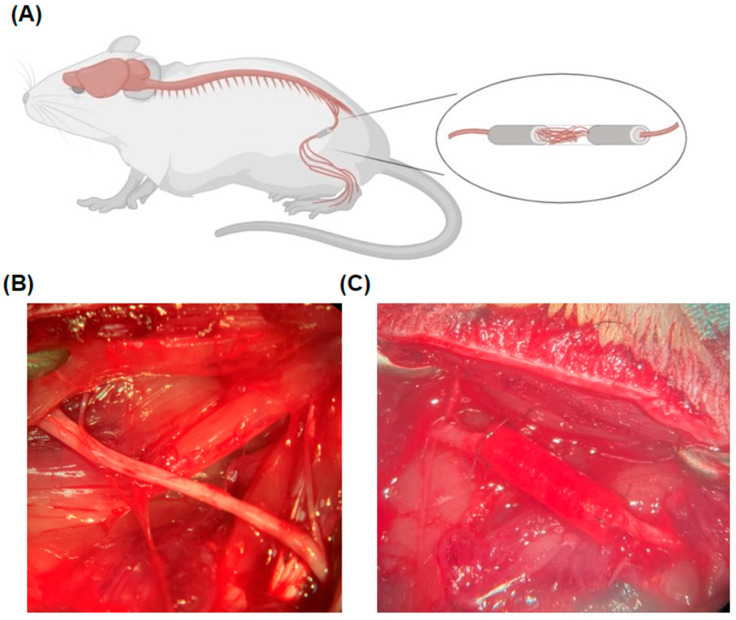
Rat sciatic nerve reconstruction. (**A**) Overview of the localization of sciatic nerve reconstruction. (**B**) Exposure of the sciatic nerve in the left hindlimb at the level of the femur. (**C**) Implantation of the conduit within the sciatic nerve following resection.

After undergoing the preoperative walking track analysis, the rats proceeded to surgery. The three groups each contained 10 rats. The groups studied were (1) an untreated conduit (UC) group serving as a negative control, (2) a group with conduits with a matrilin-2-/lysine-enhanced chitosan scaffold (TC), and (3) a reverse autograft (RA) group serving as a positive control [50]. In each rat, the left hindlimb was the operative limb, and the right hindlimb served as the control. The rats were first anesthetized with 2% isoflurane. The skin on the left thigh was thoroughly prepped, draped, and positioned under a microscope. The limb was incised to expose the left sciatic nerve. At the midline 10 mm from the trifurcation of the sciatic nerve, a 12 mm segment of the nerve was excised, with 6 mm on each side of the midline. From the proximal and distal stumps, 2 mm of each stump was drawn into a 16 mm long UC or TC such that a 12 mm gap was kept between the ends of the nerves (Figure 7). To mirror the length of the gap, the use of the reverse autograft entailed the resection of 12 mm of the sciatic nerve, flipped 180° and sutured to each end. The conduit or the autograft was reconstructed using a 10–0 nylon monofilament suture in epineural repair fashion under a microscope. The wound was closed, and the animals, after waking postoperatively, were returned to their individual cages with ad libitum access to food and water for 12 weeks, with their body weight monitored weekly. To maintain blinding, each animal was marked through coded specimen labeling using ear punching and placed into identical cages. Only the surgical team on the day of surgery had any insight into the ear punching and the random assignment into groups. The team that performed the behavioral, electrophysiologic, and histological analyses was separate from the surgical team and blinded to any of the experimental groups.

### 4.4. The Gait Analysis

**Figure 8:**
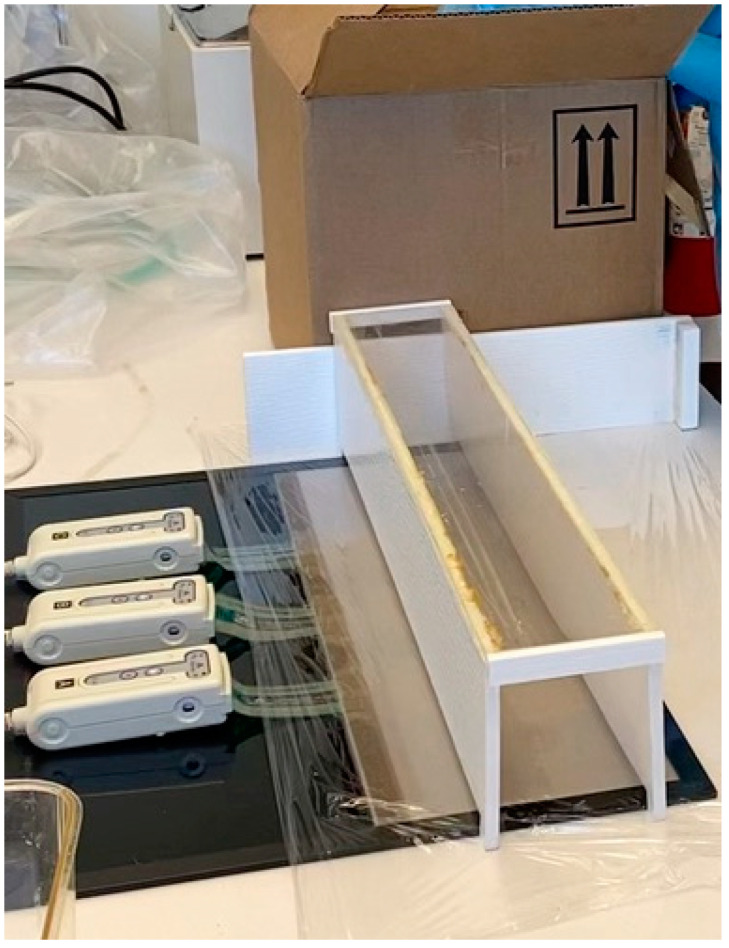
The gait testing unit comprising the Tekscan pressure sensor mat (black) that was modified to include a tinted Plexiglas tunnel (width: 17.0 cm; height: 17.0 cm; length: 44.7 cm) for the purpose of guiding the rats across the mat and into the goal box.

Prior to undergoing surgery, each rat underwent a gait pattern analysis to mark the preoperative levels of pressure it placed on its hindlimb paw. The gait patterns were analyzed using a pressure sensor mat (Tekscan VH4, Tekscan, Boston, MA, USA), which is composed of four 5101 high-resolution pressure sensor grids laid out side by side. The gait testing unit was modified to include a tinted Plexiglas tunnel (width: 17.0 cm; height: 17.0 cm; length: 44.7 cm) for the purpose of guiding the rats across the mat and ensuring that the rats remained on the sensor area during the gait trial (Figure 8). This minimized false data recordings from false steps at the edges of or outside of the sensor matrix area. Following the preoperative evaluation, the rats then underwent a gait assessment every 2 weeks after surgery over the course of the 12-week study period. A separate male Lewis rat (non-tested) was systematically put into the goal box to motivate the trial rats to run towards it. The same motivator rat was used for all animals and in all test sessions. If the animal was not motivated by the goal box, alternative positive motivators were used, such as noise and food rewards. During the data analysis, steps were automatically labeled as the right fore paw (RF), the right hind paw (RH), the left fore paw (LF), and the left hind paw (LH), in which right stood for the non-impaired side and left for the impaired side. Faulty labels caused by the intrusion of tails, whiskers, or genitalia were removed. After identification of the individual footprints, we performed an automated analysis of a wide range of parameters. The data were classified as follows: (1) individual paw statistics; (2) comparative paw statistics; (3) interlimb coordination; and (4) temporal parameters. A gait trial was considered successful only upon the completion of three runs across the full length of the Tekscan walkway [51].

### 4.5. Compound Muscle Action Potential Testing

The electrophysiological analysis was performed 12 weeks postoperatively, as previously described [52]. Electrical stimulation was applied to the nerves by placing bipolar hooked silver stimulating electrodes proximally to the conduit or the reverse autograft. The stimulating mode was set as a pulsed mode (with a 5 mA stimulus intensity, a 1 Hz frequency, and a 1 ms duration). A pair of concentric needle electrodes was inserted into the belly of the gastrocnemius muscle alongside a reference surface electrode near the distal tendon and a ground electrode in the tail. Amplification and recording were performed using a data acquisition system (Powerlab 8/35, AD Instruments Inc., Colorado Springs, CO, USA). The signals were recorded using Labchart software (AD Instruments) connected to a Bio-amplifier (Bioamp, AD Instruments, São Paulo, Brazil). The peak-to-peak amplitude and the onset latency of the CMAP were measured for the UC, TC, and RA groups. The amplitude was defined as the height by the signal level minus the baseline at the peak; the latency was defined as the time interval from the stimulus artifact to the start of the response. The measurements were all conducted at room temperature. The peak-to-peak amplitudes of the CMAP were identified for each hindlimb of the rat and reported as the proportion of the CMAP in the experimental LHL to that in the control RHL. Three separate trials were performed for each hindlimb in each rat. Immediately following the electrophysiological testing, the rats were rendered unconscious through exposure to CO_2_, and irreversible death was confirmed via thoracotomy.

### 4.6. Nerve and Muscle Extraction

Upon their sacrifice, the sciatic nerves encompassing the conduits and the reverse autografts were harvested from the rats’ left hindlimbs, as were normal sciatic nerves from the right hindlimbs. Each conduit or autograft nerve was excised with 3 mm of the native nerve stump present proximally and distally. The tibialis anterior and gastrocnemius muscles were then harvested from the tibias of both the right and left hindlimbs. These muscles were weighed as a unit and reported as the proportion of the weight in the experimental LHL to that in the control RHL for each rat.

### 4.7. Axon Analysis

Upon harvesting the sciatic nerves, they were rinsed in ice-cold PBS and were then placed into solutions of 4% paraformaldehyde to begin the fixation process at 4 C for 3 h. Following fixation, they were then rinsed in PBS for 3 × 5 min and placed into graduated concentrations of sucrose. The samples were placed into 10% sucrose for 30 min, followed by 20% sucrose for 30 min, and finally 30% sucrose for 30 min. The nerves were then separated into two categories based on their purpose. A distal 3 mm segment from the distal nerve coaptation was separated for cross-sectional axonal histomorphometry. The proximal nerve containing the conduit or the reverse autograft was then used for the immunofluorescence evaluation of Schwann cell migration and distribution. The distal 3 mm segment was then placed into a tray, filled with the Tissue-Tek O.C.T. compound, frozen with liquid nitrogen, and stored at −80 °C. Following a more edge-oriented inset location of the 3 mm segment, blocks were then carefully cross-sectioned down into 1 µM sections and stained using toluidine blue [50]. Each block area of interest provided at least three 1 µM thick sections to utilize for quantification, providing minimal overlap and specificity for axons and myelin. The transverse sections were then visualized using a digital microscope and photographed under blinding of the control and treatment groups for each slide. In ImageJ^®^, the total area of the axonal cross-section was measured by converting the pixels into micrometers via the digital scale bar. After formatting the image as 16-bit and converting the image into black and white, ImageJ’s cell counting feature was used to count the number of axons within the nerve segment by analyzing the particle count through the binary reversal of highly contrasted axons in a blinded fashion.

### 4.8. Schwann Cell Immunofluorescence

The conduits and reverse autografts from the proximal nerves were placed into trays, filled with the Tissue-Tek O.C.T. compound, flash-frozen with liquid nitrogen, and stored in −80 °C until sectioning. Cryosectioning was performed in a longitudinal fashion to understand the spatial distribution of the Schwann cells within the conduit or the graft best. Sectioning involved creating 10 µM thick sections, ensuring 5 slices were taken for staining every 200 µM through the samples. The three centrally localized, longitudinally sectioned tissues were fixed with 4% paraformaldehyde (PFA) for 3 h and then incubated with blocking solution (5% normal goat serum) for 1 h. Subsequently, the tissues were incubated overnight at 4 °C with S-100 (sc-53438, Santa Cruz Biotechnology, TX, USA) at a 1:50 ratio in an antibody dilution reagent solution (Invitrogen #003218, MA, USA). This was followed by incubation with an Alexa Fluor 594 Fluorochrome-conjugated secondary antibody for 1 h in the dark at a 1:200 ratio (Invitrogen #A11032, MA, USA) [53].The tissues were then rinsed with PBS and mounted using anti-fade media (Vectorlabs, #H-1200, CA, USA). The tissues were then observed under confocal microscopy and under blinding to the treatment group for each slide. Images were captured using a 4× objective magnification lens for DAPI and a 10× objective magnification lens for FITC. The fluorescence images were converted into 16-bit, black-and-white images. ImageJ’s fluorescence quantification system was used to count the number of DAPI- and FITC-fluorescent signals present in each image using auto color threshold optimization. The size of the average particle per area in unit^2^ was also collected through the system’s output measurements.

### 4.9. The Statistical Analysis

For each outcome, including the gait trials, CMAP, muscle weights, axon counts, and immunofluorescence assessments, a one-way ANOVA was performed given the multiple groups and the one-factor reconstructive differences noted for each using GraphPad Prism version 10.0.0 (Boston, MA, USA). A post hoc evaluation using Tukey’s multiple comparisons test was further performed to evaluate and identify significant differences between each group using GraphPad Prism version 10.0.0 (Boston, MA, USA).

## 5. Conclusions

This study highlighted the therapeutic potential of extracellular-matrix-based biomimetic nerve grafting for peripheral nerve regeneration. The utilization of a novel matrilin-2 (MATN2)- and lysine (K)-enhanced chitosan scaffold formed a multi-channel microarchitecture capable of providing a microenvironment that supported SC migration and axonal regeneration. Improved functional outcomes were observed in the walking tasks and the CMAP when applying the MATN2/K-chitosan scaffold, accompanied by a relative decrease in muscle atrophy compared to that with the use of a conduit. Our detailed histological analysis of the nerves within the MATN2/K-chitosan scaffold revealed a greater axonal number, greater SC migration, and a wider SC distribution, further supporting the regenerative biomimetic appeal of this cell-free scaffold. This work provides a basis for the use of MATN2 as an integral protein for the development of novel nerve repair strategies, along with the versatility of chitosan, of forming a readily available matrix that avoids the morbidity associated with autografts and delivers bioactivity superior to that poorly present in processed allografts. These promising results also offer intriguing future avenues for investigations centered on optimizing the nerve regeneration through enhanced study and detailing of the biomimetic activity involved within the neural extracellular matrix. While this study provides a preliminary foundation for understanding the potential of MATN2/K-chitosan scaffolds in nerve reconstruction, it also highlights the need and excitement for continued exploration and refinement in elucidating the underlying molecular mechanisms involved to optimize the development of cell-free and readily available nerve grafts for effectively reconstructing nerve injuries in a consistent manner.

## Data Availability

The data presented in this study are available on request from the corresponding authors. The authors can confirm that all of the relevant data are included in this article.

## Abbreviations

The following abbreviations are used in this manuscript:

UC	Untreated Conduit
TC	Treated Conduit
RA	Reverse Autograft
UN	Uninjured Nerve
SCs	Schwann Cells
CMAP	Compound Muscle Action Potential
MATN2	Matrilin-2
